# The relevance of leukocytosis in patients with severe trauma: a systematic review

**DOI:** 10.1186/s13037-026-00504-z

**Published:** 2026-07-23

**Authors:** Christian Thomas Hübner, Puk van Wagenveld, Felix Karl-Ludwig Klingebiel, Yannik Kalbas, Sascha Halvachizadeh, Roman Pfeifer, Hans-Christoph Pape, Michel Paul Johan Teuben

**Affiliations:** 1 Department of Traumatology, University Hospital Zurich, University of Zurich, Raemistrasse 100, Zurich, 8091 Switzerland; 2 University of Maastricht, Maastricht, The Netherlands

**Keywords:** Severe trauma, Leukocytosis, Neutrophil, Immune response, Systematic review

## Abstract

**Background:**

Polytrauma remains a leading cause of death in individuals younger than 40 years and poses major challenges during early resuscitation and subsequent clinical management. Beyond early mortality from hemorrhage, post-traumatic inflammatory and infectious complications such as systemic inflammatory response syndrome (SIRS), acute respiratory distress syndrome (ARDS), and multiple organ dysfunction syndrome (MODS) are of increasing clinical relevance. Leukocyte count is a simple and routinely available laboratory parameter. This systematic review aimed to evaluate whether leukocyte count on admission is associated with post-traumatic complications and mortality.

**Methods:**

A systematic review in accordance with the PRISMA guidelines was conducted. PubMed/MEDLINE and EMBASE were systematically searched for original English-language clinical studies published between January 1, 2000, and July 31, 2025, that investigated leukocyte counts following adult polytrauma. After deduplication, 2,169 records were independently screened by three investigators based on titles and abstracts. Thirty-two reports were sought for retrieval, and 12 studies were finally included. Disagreements were resolved by consensus. Data on study characteristics, patient and injury characteristics, leukocyte counts and kinetics, and clinical outcomes were extracted and qualitatively synthesized. Study quality and risk of bias were assessed using the Newcastle–Ottawa Scale. A sample-size-weighted mean admission leukocyte count was calculated from the reported study-level means, and associations were analyzed using Spearman’s rank correlation.

**Results:**

After subsequent screening 12 studies with overall 15,658 patients were finally included in the current review. Across all studies, leukocyte counts after trauma were elevated compared with reference values. The weighted mean leukocyte count across studies was 10.26 × 10^9/L. The proportion of blunt trauma was positively correlated with mean leukocyte count (rho = 0.775, *p* = 0.04). Pneumonia and SIRS were reported in three and four studies, respectively. One study reported higher mortality in patients with persistent leukocytosis after trauma.

**Conclusions:**

This review demonstrates that leukocyte counts are consistently elevated following trauma. Although some studies suggested associations with morbidity and mortality, the available evidence remains insufficient for firm prognostic conclusions because of substantial heterogeneity and the predominantly retrospective design of the included studies. Further research should focus on leukocyte subpopulations, particularly neutrophils, to improve understanding of post-traumatic immune responses and their clinical relevance.

**Supplementary Information:**

The online version contains supplementary material available at https://doi.org/10.1186/s13037-026-00504-z.

## Background

Severe trauma triggers an immediate and complex systemic inflammatory response, involving the activation of innate immune pathways including the release of proinflammatory cytokines and profound shifts of circulatory leukocytes [1]. This is reflected by rapid alterations of circulatory white blood cell counts, which are routinely measured as part of standard trauma assessment in most Western hospitals [2].

Leukocytosis in adults is typically defined as a peripheral leukocyte count greater than 11 × 10⁹/L [3]. Triggers of leukocytosis include infection, tissue injury, shock, ischemia and reperfusion, and stress-mediated demargination of neutrophils [4]. In the setting of trauma, leukocytosis often develops within minutes to hours after injury, largely driven by catecholamine-induced demargination and bone marrow release of neutrophils [5]. Although this response may represent an appropriate physiological reaction to injury, it has been hypothesized that excessive or persistent leukocytosis is linked to adverse outcomes, including systemic inflammatory response syndrome (SIRS), sepsis, multiple organ dysfunction, and increased mortality [6, 7].

However, the clinical significance of leukocytosis in polytrauma is not yet fully understood. The aim of this systematic review was to provide an overview of post-traumatic leukocyte alterations and their potential influence on the occurrence of morbidity and mortality. This is of particular interest, since leukocyte count is a simple and widely available biomarker, but currently leukocytosis is not incorporated into standard trauma prognostic models, and its utility for early risk stratification in polytrauma remains uncertain.

## Methods

This systematic review was conducted in accordance with the Preferred Reporting Items for Systematic Reviews and Meta-Analyses (PRISMA) guidelines (http://www.prisma-statement.org/). A systematic review was conducted to identify all relevant original clinical publications analyzing leukocyte counts after polytrauma. Only original articles written in English were included in order to minimize the heterogeneity of the analysis and to optimize the quality of included datasets. Studies on pediatric patients, experimental studies, animal trials, case reports, case series, reviews and editorials were excluded.

The PubMed/MEDLINE and EMBASE databases were searched to cover the period from January 1, 2000, to July 31, 2025. The search strategy included a combination of controlled vocabulary terms (MeSH/Emtree terms) and regular search terms, connected by Boolean operators. Truncation was applied to account for plural forms and alternate spellings, all relevant synonyms were included. Filters were applied to exclude inappropriate article types. The full list of search terms is provided in Supplementary File 1. The search results were organized and deduplicated using Clarivate™ EndNote™ version 20.

**Fig. 1:**
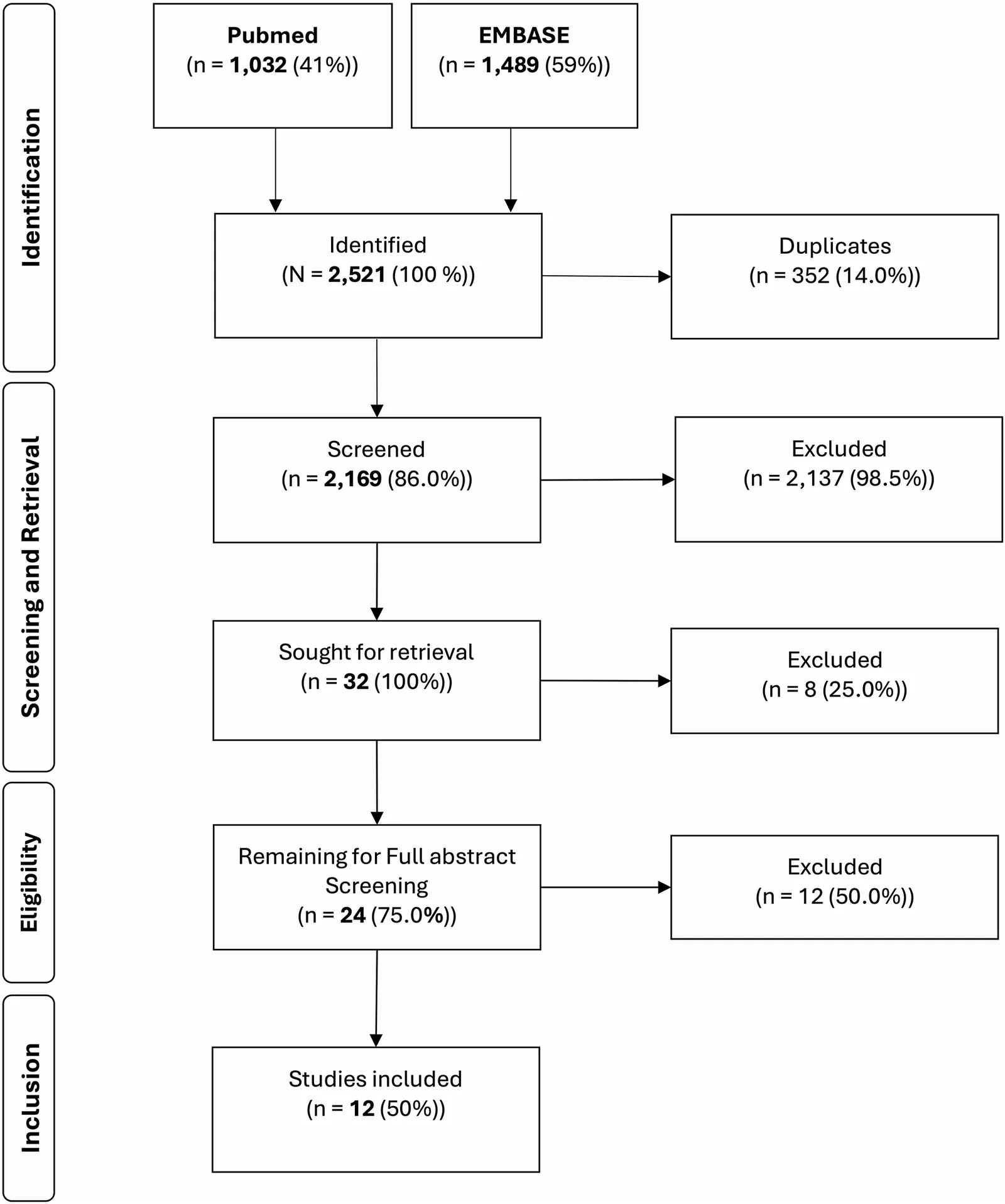
Flowchart of the selection process

PvW, CTH, and FKLK independently screened the titles and abstracts of the identified articles. CTH and PvW cross-checked the extracted data and resolved any disagreements through personal discussion. Full texts of potentially eligible studies were retrieved through the university’s central library via the respective publishers. Figure 1 provides a flowchart of the selection process. Study quality and risk of bias were assessed using the Newcastle–Ottawa Scale (NOS) for cohort studies [8]. Studies were categorized as high quality (7–9 points), moderate quality (5–6 points), or low quality (< 5 points).

Information reported regarding alterations (increase or decrease) in leukocyte count following polytrauma was extracted from the included studies. Additionally, patient and trauma characteristics and reported clinical outcomes were grouped and analyzed using a qualitative approach. Furthermore, time windows and study characteristics were reported.

Statistical analyses were performed using R-Studio (R Foundation for Statistical Computing, Vienna, Austria, version 2024.12.1 + 563). Graphs were generated using GraphPad Prism version 10.7.0 for macOS (GraphPad Software, San Diego, California, USA). Correlation analysis was performed using the Spearman’s rank-correlation test. The Cohen-classification was used to determine the level of correlation [9].

## Results

A total of 2,169 publications were identified and screened for eligibility. Of those, 12 retrospective cohort studies published between 2003 and 2025 were included. The studies were conducted in the United States of America (*n* = 6), the Netherlands (*n* = 2), Switzerland (*n* = 2), Turkey (*n* = 1) and Mexico (*n* = 1), mostly in level 1 trauma centers. Sample sizes ranged from 51 to 5,950 patients. Data from a total of 15,658 patients were analyzed across all included studies. Table 1 provides an overview of the selected studies.

**Table 1:** Study characteristics of selected studies

Nr.	Authors	Title	Year of publication	Country	Journal	Sample size
1	Cander et al. [10]	Morphologic and quantitative analyses of leukocytes in patients with multiple system trauma	2007	Turkey	Advances in Therapy	51
2	Chang et al. [11]	Early leukocytosis in trauma patients: what difference does it make?	2003	United States of America	Current Surgery	786
3	De Dios Díaz-Rosales et al. [12]	Leukocytosis in penetrating trauma (abdominal and osseous/thoracic): a cross sectional study in Juárez, México	2013	Mexico	Archivos de Medicina	186
4	Hasjim et al. [13]	Moderate to severe leukocytosis with vasopressor use is associated with increased mortality in trauma patients	2022	United States of America	Journal of the Intensive Care Society	332
5	Heffernan et al. [7]	Failure to normalize lymphopenia following trauma is associated with increased mortality, independent of the leukocytosis pattern	2012	United States of America	Critical Care	2,448
6	Jol et al. [14]	Similar change in platelets and leucocytes 24 h after injury is associated with septic shock a week later	2017	The Netherlands	ANZ Journal of Surgery	41
7	Niggli et al. [15]	Sex differences in the time trends of sepsis biomarkers following polytrauma	2025	Switzerland	Scientific Reports	3,059
8	Paladino et al. [16]	Leukocytosis as prognostic indicator of major injury	2010	United States of America	Western Journal of Emergency Medicine	805
9	Santucci et al. [17]	Leukocytosis as a predictor of severe injury in blunt trauma	2008	United States of America	Western Journal of Emergency Medicine	279
10	Schnüriger et al. [18]	Serial white blood cell counts in trauma: do they predict a hollow viscus injury?	2010	United States of America	The Journal of Trauma	5,950
11	Teuben et al. [19]	Splenectomy is associated with altered leukocyte kinetics after severe trauma	2021	The Netherlands	European Journal of Medical Research	129
12	Teuben et al. [20]	The 24-Hour Leukocyte Gap as a novel predictor for sepsis in adult severe blunt trauma	2025	Switzerland	Langenbecks Archives of Surgery	1,592

### Study quality and risk of bias analysis

The methodological quality of the included studies, measured by the NOS score, ranged from 4 to 9 points. Six studies were classified as high quality (7–9 points), three as moderate quality (5–6 points), and two as low quality (< 5 points). The mean NOS score across all included studies was 7.0 points (± 1.7). With regard to individual domains, the mean score for selection was 2.9 out of 4 points, for comparability 1.6 out of 2 points, and for outcome 2.5 out of 3 points. A detailed item-level assessment is provided in Supplementary Table S1.

### Demographic data

Across the studies, most patients were male, with a pooled mean of 74.5% (Range 59% to 90%), and generally in the young to middle-adult age range (pooled mean age 40.4 years). Blunt trauma was the predominant mechanism of injury, accounting for 80.7% of all cases, however, most studies also reported a low percentage of penetrating trauma. One study analyzed exclusively penetrating trauma [12]. Mean Injury Severity Score (ISS), Glasgow Coma Score (GCS), systolic blood pressure (SBP) and pulse rate (PR) were inconsistently reported across the studies. In seven studies ISS was reported [7, 13–15, 18–20]. The majority reported patients with major trauma (ISS > 15). A pooled mean ISS of 18.8 across all studies was calculated. Mean GCS was reported in five studies and ranged from 9 to 14 [13, 15, 18–20]. In four studies, mean SBP was reported and ranged from 120 to 138mmHg, as well as pulse ranging from 89 to 103/min [13, 16, 19, 20]. Table 2 provides an overview of the patient demographics.

**Table 2:** overview of patient demographics

Nr.	Female	Male	Age (Range)	Age (mean)	ISS (Mean)	GCS (Mean)	SBP (Mean)	PR (Mean)	Mechanism of injury	
									Blunt	Penetrating
1	41%	59%	16–59	n.a.	n.a.	n.a.	n.a.	n.a.	n.a.	n.a.
2	23%	77%	> 14	n.a.	n.a.	n.a.	n.a.	n.a.	58%	42%
3	10%	90%	14–65	29	n.a.	n.a.	n.a.	n.a.	0%	100%
4	27%	73%	> 17	31	24	15	128	103	84%	16%
5	31%	69%	> 17	53	23	n.a.	n.a.	n.a.	94%	6%
6	20%	80%	16–80	44	28	n.a.	n.a.	n.a.	95%	5%
7	26%	74%	> 15	43	27	12	n.a.	n.a.	92%	8%
8	25%	75%	13–95	39	n.a.	n.a.	138	89	46%	34%
9	37%	63%	0–75+	n.a.	n.a.	n.a.	n.a.	n.a.	100%	0%
10	23%	77%	> 16	34	10	14	n.a.	n.a.	74%	26%
11	29%	71%	> 17	34	29	14	120	90	n.a.	n.a.
12	26%	74%	> 17	44	27	9	132	90	100%	0%

### Leukocyte counts

Systematic leukocyte counts at admission were reported in most, but not all, studies. Mean values at admission ranged between 9.2 × 10^9/L and 24.1 × 10^9/L, with a weighted mean of 10.26 × 10^9/L. Further timepoints of leukocyte measurements were inconsistent in the literature. The most commonly additional timepoints were 24 h and 48 h. Three studies measured long term leukocyte counts [14, 15, 19], however the data were only partially presented in these studies. Two studies did not even provide leukocyte counts after admission [7, 14]. Table 3 gives an overview of the leukocyte counts.

**Table 3:** Overview of leukocyte counts and patient outcomes

Nr.	Leukocyte count on admission (x10^9/L, Mean)	Further leukocyte counts	Mortality	Hospital length of stay	ICU length of stay	Pneumonia	Sepsis
1	14.4	None	n.a.	n.a.	n.a.	n.a.	n.a.
2	10	None	2.8%	n.a.	n.a.	n.a.	n.a.
3	15.1	Pre-operatively	n.a.	9.5 days	n.a.	n.a.	n.a.
4	24.1	Daily	14.5%	11.7 days	4.2 days	22%	11.7%
5	n.a.	None	n.a.	31.1 days	3.9 days	n.a.	n.a.
6	n.a.	At 18 h, 24 h, 2d, 3d, 4d, 5d, 6d, 7d, 8d, 9d, 10d, 11d, 12d, 13d and 14d	7.3%	n.a.	17 days	n.a.	n.a.
7	12.3	At 1 h, 2 h, 3 h, 4 h, 6 h, 8 h, 12 h, 24 h, 48 h, 3d, 4d, 5d, 7d, 10d, 14d and 21d	31.0%	13 days	5 days	20.1%	17%
8	9.2	None	n.a.	n.a.	n.a.	n.a.	n.a.
9	14	None	n.a.	n.a.	n.a.	5%	n.a.
10	11.6	At 24 h	n.a.	n.a.	n.a.	n.a.	n.a.
11	15.8	At 6 h, 16 h, 2d, 3d, 4d, 5d or 6d, 7d and 14d	4.7%	15.9 days	3.2 days	23.3%	n.a.
12	13.4	At 24 h	10.7%	22.8 days	11.9 days	n.a.	15.8%

### Leukocyte kinetics and clinical outcome

Mortality has been reported in six studies, with rates ranging from 2.8% to 31.0% [11, 13–15, 19, 20]. Hospital length of stay was described in six studies [7, 12, 13, 15, 19, 20], varying between 9.5 and 22.8 days, while ICU length of stay was reported in six studies, ranging from 3.2 to 17 days [7, 13–15, 19, 20]. Pneumonia was reported in four studies, occurring in 5% to 23.3% of patients [13, 15, 17, 19], and sepsis was reported in three studies, with rates of 11.7%, 15.8%, and 17% [13, 15, 20]. However, in most of the studies outcome parameters were not predefined as endpoints. One study demonstrated higher mortality among patients with severe leukocytosis at admission [13] and two studies associated persisting leukocytosis with higher mortality rates [7, 20]. One study reported increased rates of sepsis and infectious complications, depending on leukocytosis [14]. An overview of the reported outcomes is provided in Table 3.

### Leukocyte count and percentage of blunt trauma

**Fig. 2:**
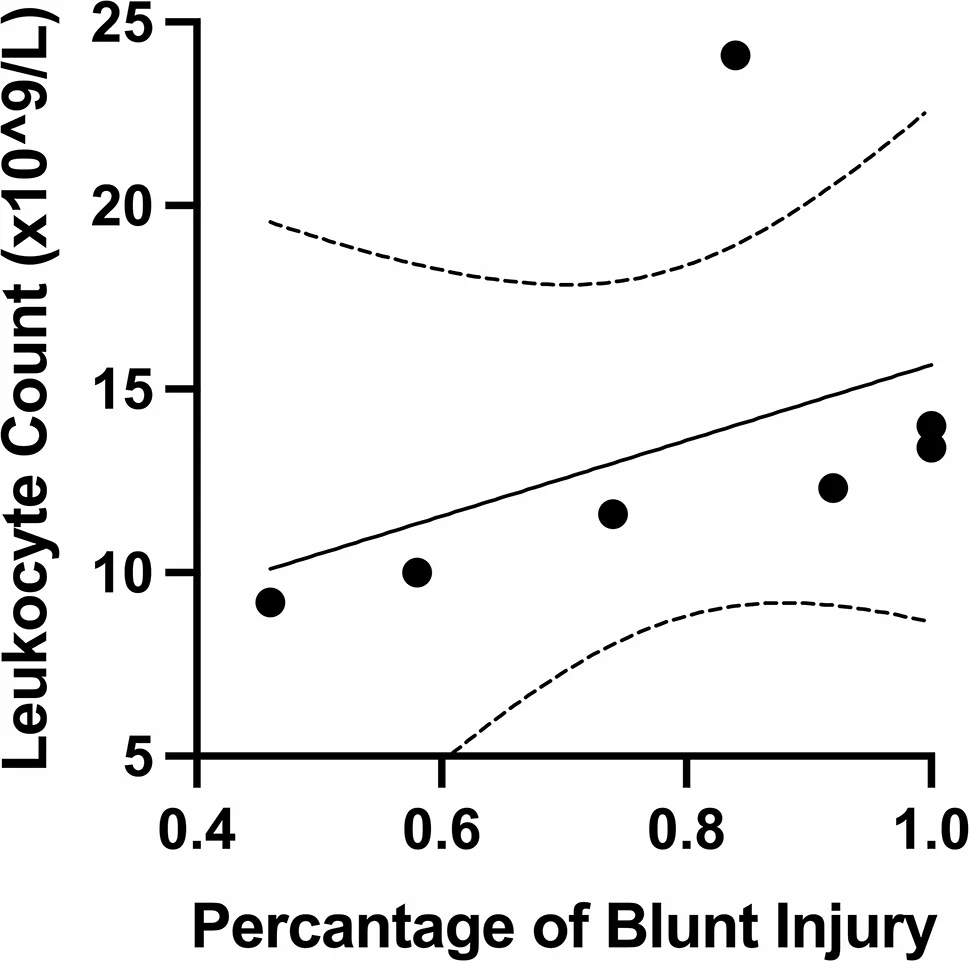
Correlation between the proportion of blunt trauma and mean admission leukocyte count

Studies with a higher proportion of blunt trauma generally reported higher admission leukocyte counts compared to those with more penetrating injuries. The proportion of blunt trauma correlated significantly with mean admission leukocyte counts (Spearman’s rank coefficient ρ = 0.775, *p* = 0.04). Figure 2 illustrates the distribution of blunt trauma and its correlation with leukocyte count.

## Discussion

Leukocytosis after polytrauma is frequently observed and can easily be detected through routine blood sampling. Despite this, literature on the significance of this finding is scarce. In this systematic literature review, we identified 12 studies examining leukocyte dynamics following trauma, all of which reported elevated leukocyte counts immediately after injury, particularly in patients with severe trauma.

Blunt trauma emerged as a key driver of post-traumatic leukocytosis. The observed strong correlation between the proportion of blunt injuries and higher admission leukocyte counts is consistent with recent literature on enhanced neutrophil mobilization from the bone marrow into circulation after blunt injury and major visceral surgery [21, 22].

Several studies have attempted to define a correlation between leukocytosis and injury severity, as well as clinical outcomes, such as mortality and infectious complications. One study found that ISS greater than 15 and GCS less than 9 were associated with increased leukocyte counts [11]. Another study showed that leukocytosis, particularly when persistent or accompanied by vasopressor use, appears to predict higher mortality and sepsis risk [13]. It is tempting to hypothesize that vasopressors contribute to the leukocytosis by stimulating bone marrow cell mobilization. Mortality rates rose with leukocytosis severity, suggesting it may reflect physiological deterioration rather than serve as an isolated marker. Other studies reported only weak correlations between major injury (ISS > 15) and leukocyte count elevation, thus failing to demonstrate consistent predictive value of leukocyte count for sepsis or death [17, 18]. A limitation of the included studies is that the majority of them did not report outcome parameters as predefined endpoints. This makes it difficult to draw conclusions from the studies or to perform a meta-analysis. However, the limited discriminatory power of single leukocyte measurements and the lack of differentiation into subpopulations of white blood cells likely explain the inconsistent predictive value across studies.

One important aspect that was only addressed to a limited extent in the included studies was the association between leukocytosis and injury pattern. Thoracic injury, in particular, is known to contribute to leukocytosis and neutrophil activation after polytrauma [23]. One study included in this review described higher WBC counts in patients with thoracic injuries compared with patients with isolated abdominal injuries [12]. Leukocytosis in these patients might be induced by the release of cells sequestered in the lungs after trauma, as well as by the mobilization of leukocytes from the bone marrow into the circulation due to the release of damage-associated molecular patterns (DAMPs) following lung injury. Both mechanisms may contribute to a higher susceptibility to infectious complications, pneumonia, and multiple organ dysfunction syndrome (MODS) [24, 25].

Furthermore, the spleen, as an immunologic organ, is of special interest and splenic injury is associated with higher WBC counts [11]. Studies investigating the dynamics of WBC after splenectomy, reveal elevated WBC counts in patients undergoing splenectomy [19, 26].

The temporal pattern of leukocytosis after trauma may provide further insights into the posttraumatic immune response. Early transient leukocytosis is considered as part of the physiological stress response to trauma, resulting from catecholamine-induced demargination and rapid bone marrow mobilization [27]. By contrast, persistent leukocytosis or failure of leukocyte counts to normalize has been associated with sepsis, infectious complications and mortality [14]. One study established the 24 h-leukocyte gap as a new prognostic parameter for sepsis and infectious complications [20].

Interestingly, also admission leukocytosis seems to predict a higher incidence of infectious complications, including pneumonia and sepsis [14, 15, 20]. Although none of the included studies differentiated between leukocyte subpopulations, it is likely that the observed elevations are primarily driven by neutrophils as these cells account for > 90% of circulatory white blood cells after trauma [28]. These cells may be rapidly mobilized from the bone marrow into circulation after trauma and have a relevant proportion in the composition of leukocytosis after trauma. Especially immature cells, freshly released from the bone marrow, are thought to have limited immune functional capacities [29].

## Conclusion

Leukocytosis after polytrauma is a simple and rapidly measurable marker, reflecting the early systemic inflammatory reaction to injury. This systematic review shows that more severe trauma is associated with the occurrence of post-traumatic leukocytosis. Furthermore, the occurrence or inability to recover promptly from post-traumatic leukocytosis is associated with impaired outcome, although not in all studies.

Future prospective multicenter studies on homogenous populations should focus on leukocyte subpopulations, such as neutrophils and their post-insult kinetics. This may provide more meaningful insight into immune dynamics after trauma and improve risk stratification for critically injured patients.

## Supplementary Information

Below is the link to the electronic supplementary material.

Supplementary Material 1

Supplementary Material 2

## Data Availability

The datasets used and/or analyzed during the current study are available from the corresponding author on reasonable request.
