# Supplementary material for: The relevance of leukocytosis in patients with severe trauma: a systematic review
Source: Patient Saf Surg. 2026 Jul 23;20:39. doi: 10.1186/s13037-026-00504-z (PMC13685343; doi:10.1186/s13037-026-00504-z)
Supplement: Supplementary file 1 [file 13037_2026_504_MOESM1_ESM.docx]

Leukocytosis after severe Trauma

P: Patients
E : (Poly)Trauma
C: -
O: Leukocytosis, Leukopenia, Leukocyte count

PUBMED

("Multiple Trauma"[MeSH Terms] OR "fractures, bone"[MeSH Terms] OR "fractur*"[Title/Abstract] OR "trauma*"[Title/Abstract] OR "polytrauma"[Title/Abstract] OR "polytraum*"[Title/Abstract])

**AND**

"Leukocytosis"[Mesh] OR "Leukopenia"[Mesh] OR "Leukocyte Count"[Mesh] OR “Leukocytosis"[Title/Abstract] OR “Leukopenia"[Title/Abstract]

**AND**

("2000/01/01"[PDAT] : "2025/07/31"[PDAT])

- 1032 results PUBMED

EMBASE

'multiple trauma'/mj OR 'injury'/mj OR 'polytrauma':ab,ti OR 'polytraum*':ab,ti OR 'fractur*':ab,ti OR 'trauma*':ab,ti

**AND**

'leukocytosis'/mj OR 'leukopenia'/mj OR 'leukocyte count'/mj OR 'leukocytosis':ab,ti OR 'leukopenia':ab,ti

**AND**

[2000-2025]/py

- Results EMBASE: 1489

Imported in Endnote: 1032 + 1489 =2521

Duplicates removed in Endnote: 352

Remaining for Rayyan import: 2169

Search performed: 06.08.2025
