# Supplementary material for: The relevance of leukocytosis in patients with severe trauma: a systematic review
Source: Patient Saf Surg. 2026 Jul 23;20:39. doi: 10.1186/s13037-026-00504-z (PMC13685343; doi:10.1186/s13037-026-00504-z)
Supplement: Supplementary file 2 [file 13037_2026_504_MOESM2_ESM.docx]

**Supplementary Table S1: Risk of bias assessment using Newcastle-Ottawa Scale (NOS)**

| **Nr.** | **Study** | **Selection (0–4)** | **Comparability (0–2)** | **Outcome (0–3)** | **Total (0–9)** | **Quality** |
| --- | --- | --- | --- | --- | --- | --- |
| 1 | Cander et al., 2007 [24] | 2 | 0 | 2 | 4 | Low |
| 2 | Chang et al., 2003 [18] | 4 | 2 | 3 | 9 | High |
| 3 | De Dios Díaz-Rosales et al., 2013 [10] | 2 | 0 | 2 | 4 | Low |
| 4 | Hasjim et al., 2022[11] | 3 | 2 | 3 | 8 | High |
| 5 | Heffernan et al., 2012 [7] | 3 | 2 | 3 | 8 | High |
| 6 | Jol et al., 2017 [12] | 2 | 1 | 2 | 5 | Moderate |
| 7 | Niggli et al., 2025 [13] | 3 | 2 | 3 | 8 | High |
| 8 | Paladino et al., 2010 [17] | 3 | 1 | 2 | 6 | Moderate |
| 9 | Santucci et al., 2008 [19] | 3 | 1 | 2 | 6 | Moderate |
| 10 | Schnüriger et al., 2010 [14] | 3 | 2 | 3 | 8 | High |
| 11 | Teuben et al., 2021 [16] | 3 | 2 | 3 | 8 | High |
| 12 | Teuben et al., 2025 [15] | 4 | 2 | 3 | 9 | High |

Studies were categorized as high (7–9), moderate (5–6), or low (<5) quality.
